# Serotype 3 *Streptococcus pneumoniae* Escapes the Immune Responses Induced by PCV13 in Mice With High Susceptibility to Infection

**DOI:** 10.1002/iid3.70062

**Published:** 2024-12-06

**Authors:** Giuliana S. Oliveira, Johanna Rivera, Tasson C. Rodrigues, Giovanna B. Carneiro, Orlando G. Ribeiro, Eliane N. Miyaji, Liise‐anne Pirofski, Maria Leonor S. Oliveira

**Affiliations:** ^1^ Bacteriology Laboratory Instituto Butantan São Paulo Brazil; ^2^ Division of Infectious Diseases, Department of Medicine Albert Einstein College of Medicine and Montefiore Medical Center New York Bronx USA; ^3^ Immunogenetics Laboratory Instituto Butantan São Paulo Brazil

## Abstract

**Background:**

*Streptococcus pneumoniae* (pneumococcus) is a common cause of respiratory and invasive infections in humans. PCV13, a pneumococcal conjugate vaccine used globally, is highly effective against diseases caused by pneumococcal serotypes included in its formulation. However, one of them, the serotype 3 (ST3) is still being relatively commonly isolated from patients, suggesting an escape from vaccine‐induced immunity. The thick capsule produced by ST3 facilitates bacterial evasion from the immune system. Additionally, host immune responses may influence the outcome of ST3 infection. Here we evaluated the influence of inflammation in the adaptive immune responses and protection induced by PCV13 against ST3, using two outbred mice lines that were phenotypically selected for high (AIRmax) and low (AIRmin) inflammatory responses.

**Methods:**

AIRmin and AIRmax mice were immunized with PCV13. Inbred BALB/c mice were used as reference for vaccine efficacy. Induction of IgG against polysaccharides (PS) from pneumococcal serotype 1 (ST1) and ST3 were evaluated by ELISA. Protection was tested against invasive infections with ST1 and ST3 pneumococcal strains. Sera were compared by IgG binding to pneumococcal surface, induction of pneumococcal agglutination and opsonophagocytosis. The phagocytic capacity of mice‐derived neutrophils was also evaluated.

**Results:**

Immunization of AIRmin, AIRmax and BALB/c mice with PCV13 induced IgG against PS from ST1 and ST3 pneumococci. Despite vaccination, AIRmin mice were not protected against fatal infection with ST3. Sera from AIRmin mice immunized with PCV13 presented lower levels of anti‐PS3 IgG, with reduced capacity to bind to pneumococcal surface. Reduced capacity to induce opsonophagocytosis of ST3 pneumococci in vitro was also observed. Conversely, PCV13 protected AIRmin mice against fatal infection with ST1 and this correlated with the capacity of the sera to induce ST1 opsonophagocytosis.

**Conclusions:**

Our results show that both host and bacterial features can influence the outcome of protection induced by PCV13 against ST3 pneumococcal infection.

## Graphical abstract

ST3 evasion from PCV13‐induced immune responses Antibody responses to ST3 pneumococci

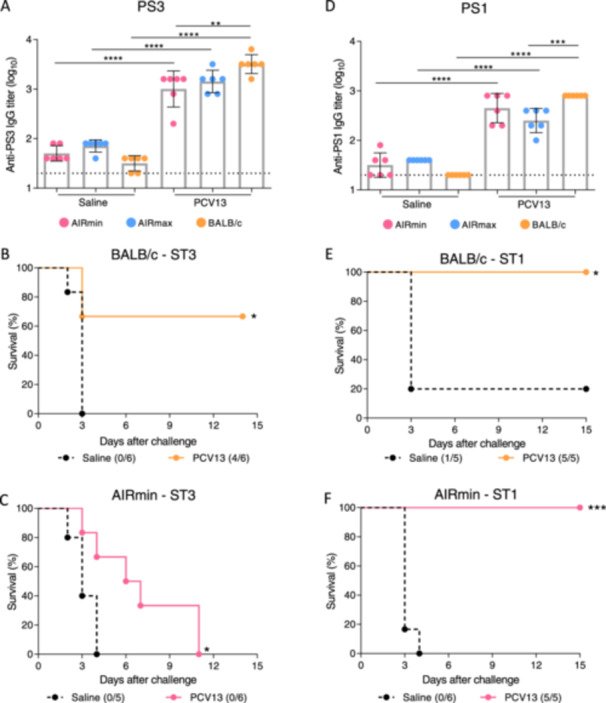

## 1 Introduction

*Streptococcus pneumoniae* (pneumococcus) colonizes the nasopharynx of healthy individuals but can also cause respiratory and systemic infections [1]. Pneumococcal diseases (pneumonia, otitis media, meningitis, sepsis), were responsible for over 800,000 deaths in 2019, among which, 340,000 were in children under 5 years old [2]. The occurrence of pneumococcal disease is influenced by both host conditions and bacterial characteristics [3, 4]. Host pro‐inflammatory innate responses are triggered by pneumococcal infection and participate in bacterial clearance [5, 6]. However, the quality and intensity of the inflammatory response can be influenced by factors such as age, comorbidities and previous infections with respiratory viruses, which affect pneumococcal colonization and disease development [7, 8, 9]. On the other hand, pneumococci display a high capacity for transformation, resulting in antigenic variability, as well as a phase‐variable control of antigen expression, which contributes to the evasion from the immune system [10, 11].

The main pneumococcal virulence factor is the polysaccharide (PS) capsule, which helps bacteria to evade phagocytosis by host cells [12]. Structural and antigenic variability of the capsular PS, drives the classification of pneumococci into more than 100 serotypes (ST) described to date [13, 14]. Pneumococcal conjugate vaccines (PCVs) are composed of PS from the bacterial capsule conjugated to carrier proteins and provide protection through the induction of anti‐PS antibodies that opsonize bacteria promoting phagocytosis and killing by immune cells. PCV formulations differ in the number of PS from prevalent disease‐causing pneumococcal STs. These vaccines are highly effective against nasopharyngeal colonization and invasive pneumococcal disease (IPD) caused by the ST present in the formulations, also referred to as vaccine‐types.

Pneumococcal ST3 is highly associated with IPD and mortality [15]. The introduction of PCV13 (Prevnar, Pfizer), which contains PS from ST3 (PS3), significantly reduced the occurrence of ST3‐related diseases in several countries [16, 17]. Despite this reduction, ST3 still appears as an important cause of IPD [18, 19]. Therefore, studies that address the relationship between vaccine‐induced immune responses and protection against ST3 can contribute to the development of more appropriate prophylactic and therapeutic interventions.

Animal models of pneumococcal infections have contributed to the study of host‐pathogens interactions and the mechanisms of protection induced by vaccines [3, 20, 21, 22, 23]. We previously evaluated the influence of inflammation in innate immune responses to pneumococcal infection in two outbred mice lines that were phenotypically selected for minimal (AIRmin) and maximal (AIRmax) acute inflammatory responses [24, 25]. The high inflammatory status of AIRmax mice results in a profile of resistance against pneumococcal infections by different serotypes, including ST3, whereas AIRmin mice are highly susceptible [25]. Similarly to AIRmax, AIRmin mice secreted several pro‐inflammatory cytokines and chemokines in the respiratory tract upon ST3 infection. However, AIRmin mice were not able to clear bacteria from the lungs, resulting in the dissemination to the bloodstream and the development of disease. Decreased levels of the CXCL5 chemokine and a lower influx of neutrophils, at the site of infection, was observed when compared with AIRmax mice. In addition, higher percentages of neutrophils in apoptosis and necrosis, after ST3 infection, were also observed in AIRmin mice [25]. These results suggest a profile of innate immune response that results in lower neutrophils at the site of infection and lead to increased susceptibility to pneumococci in this model.

The characterization of immune response to vaccines in conditions of increased susceptibility to pneumococcal infections can contribute to the development of prophylactic and therapeutic proposals directed to individuals that present such conditions. Here, we aimed to characterize the adaptive immune responses and protection against pneumococcal infections, especially against ST3, induced by the PCV13 vaccine in AIRmin mice. Antibody responses as well as neutrophils phagocytic capacity were evaluated. The study was extended to AIRmax mice, as a counterpart for inflammatory acute responses and BALB/c mice as a reference for vaccine‐induced protection.

## 2 Methods

### 2.1 Ethics Statement

This study was performed according to the guidelines outlined by the Brazilian National Council for Control of Animal Experimentation (CONCEA), consonant with international guidelines for animal welfare, the ARRIVE guidelines and the principles of 3Rs. Experimental protocols were approved by the Ethic Committee on Animal Use of the Butantan Institute (CEUAIB) under protocol numbers 8899030217 and 3963061221. Male and female (6‐8‐weeks‐old, matched for gender) outbred AIRmin and AIRmax (Ibut:AIRL and Ibut:AIRH, ILAR, Institute for Laboratory Animal Research, National Research Council, Washington DC, USA) mice were produced by the Immunogenetics Laboratory of Butantan Institute (Sao Paulo – Brazil). Female SPF BALB/c mice (6‐8‐weeks old) were produced by the Medical School of the University of Sao Paulo (Sao Paulo – Brazil). Animals were housed in a ventilated cabinet, located in a BSL‐2 facility, under controlled temperature and light cycle (12/12 h, light/dark cycle) with daily monitoring. After arrival, mice were randomized in the groups in a blinded way and maintained for 1 week for acclimatation, before starting procedures. Food and water were given ad libitum. Monitoring and manipulation were done by trained personnel.

### 2.2 Bacteria and Growth Conditions

*Streptococcus pneumoniae* strains 3JYP2670 (ST3), A66.1 (ST3) and ATCC6301 (ST1) were grown on blood agar for 18 h at 37°C. Liquid cultures were performed in Todd‐Hewitt medium (Difco, Beckton Dickinson, USA) containing 0.5% of yeast extract (THY) at 37°C, without agitation, until mid‐exponential phase (OD_600_ = 0.4). Frozen stocks were maintained at −80°C in THY containing 20% glycerol.

### 2.3 Animal Immunization and Pneumococcal Challenges

Mice (4–6 per group, depending on the experiment) were immunized with 1/10 of the human dose of PCV13 (PREVNAR 13, Pfizer Inc., USA) and control groups received saline. Immunization was performed with three doses, fifteen days apart, through the subcutaneous route, in a total volume of 100 µL. Blood was collected before (pre‐immune) or fourteen days after the last immunization via the retroorbital plexus under local anesthesia with proxymetacaine chloride (Alcon, USA).

Pneumococcal challenges were performed 21 days after the last dose. Mice (5–6 per group) were anesthetized intraperitoneally with a mixture of 20 mg/kg of xylazine hydrochloride and 100 mg/kg of ketamine hydrochloride (CEVA, Brazil) and then inoculated nasally with 3 × 10^5^ colony forming units (CFU) of the 3JYP2670 strain or 1 × 10^6^ CFU of the ATCC6301 strain diluted in 50 µL saline. Survival was assessed for up to 15 days. All animals were monitored daily and scored according to the health status. Animals that presented low activity due to disease progression were immediately euthanized with a lethal dose of anesthetics (60 mg/kg of xylazine hydrochloride and 300 mg/kg of ketamine hydrochloride). In some experiments, animals (four per group) were euthanized at 12 h or 24 h after the challenge, for collection of bronchoalveolar lavage fluids (BALFs), as previously described [26]. Aliquots were plated on blood agar for CFU counting. BALFs were centrifuged at 100 g for 10 min at 8°C and cells were used for the analysis of neutrophils by flow cytometry, using anti‐Ly6G ‐ APC‐Cy7 (BD Biosciences, clone 1A8) and anti‐CD11b – PerCP Cy5.5 (BD Biosciences, clone M1/70) antibodies, in 1:100 dilutions. Cells were evaluated in a FACS Canto II equipment (BD Biosciences) with 30,000 events recorded. Flow cytometry results were analyzed using the FlowJo V10.1 software (FlowJo, LLC, USA).

### 2.4 ELISA Assays

The induction of antibodies against purified PS3 and PS from ST1 (PS1) (American Type Culture Collection, USA) pneumococci was evaluated by ELISA, as previously described [27]. Briefly, sera from immunized mice were incubated with purified non‐vaccine PS from ST22F pneumococci (5 µg/mL) for 30 min at room temperature. Samples were transferred to 96‐well plates coated with 10 µg/mL with purified capsular PS3 or PS1 and serially diluted. Horseradish peroxidase conjugate sheep anti‐mouse IgG (Sigma Aldrich, USA) was used as secondary antibody. Reactions were developed with OPD (o‐Phenylenediamine dihydrochloride) substrate and the absorbance was measured at 492 nm in an EZ microplate reader 400 (Biochrom, USA) equipment. Titers were defined as the reciprocal of the sera dilutions that produced a reading of A_492nm_ = 0.1. Results were confirmed using sera from at least three independent immunization experiments.

### 2.5 Binding of Antibodies on Pneumococcal Surface

The antibody binding assay was performed for the 3JYP2670 and ATCC6301 strains as previously described [28]. Briefly, bacteria were incubated with heat‐inactivated pooled sera from each experimental group (6 mice per group) in a concentration of 1% or 5% (V/V), depending on the experiment, for 30 min on ice. Samples were washed and goat anti‐mouse IgG conjugated with fluorescein isothiocyanate (FITC) (MP Biomedicals, USA) was added to the mixtures (1:100) and incubated for 30 min on ice. Samples washed, fixed with 100 μL of Cytofix (BD Biosciences, California, USA) and analyzed by flow cytometry using FACS Canto II, with 15,000 gated events recorded. FITC‐Mean Fluorescence Intensity (MFI) for each curve was analyzed using the FlowJo V10.1 software. Results were obtained with sera from two immunization experiments.

### 2.6 Pneumococcal Agglutination Assay

The agglutination assay was performed as previously described [29]. Briefly, 10^7^ CFU of the 3JYP2670 or A66.1 pneumococcal strains were incubated with different concentrations of heat‐inactivated pooled sera from 6 mice immunized with PCV13, for 1 h at 37°C in a final volume of 50 μL. Preimmune sera were used as controls. Bacteria were analyzed by flow cytometry based on their size (forward scatter ‐ FSC) and granularity (side scatter ‐ SSC). The acquisition of 200,000 events was carried out in a FACS Canto II equipment. Results were analyzed using the FlowJo V10.1 software. Results were obtained with sera from two immunization experiments. Quantification of pneumococcal agglutinates was performed at a concentration of 40% sera, using three independent pools (three animals per pool).

### 2.7 Opsonophagocytic Assay

The opsonophagocytic assay (OPKA) was based on the reference protocol for testing opsonic capacity of pneumococci by sera [30] with modifications. J774 (mouse reticulum sarcoma‐derived macrophage cell line) were cultured in in Dulbecco's Modified Eagle Medium (DMEM, Gibco, USA) containing 10% FCS (Gibco) and 1% Pen Strep (Gibco), at 37°C, in 5% CO_2_. Cell suspensions were washed once with Hanks Balanced Salt Solution (HBSS, Gibco) without Ca^2+^ and Mg^2+^ and once with HBSS with Ca^2+^ and Mg^2+^, then, suspended in Opsonization Buffer B (OBB ‐ 10% gelatin, 5.4% fetal bovine serum in HBSS with Ca^2+^ and Mg^2+^). Aliquots containing 10^3^ bacteria were added to 96‐well plates and incubated for 30 min at room temperature. Then 4 × 10^5^ J774 cells were added to the wells (to get a phagocyte/bacteria ratio of 400:1), along with 6.25 μL of sera from naïve SPF BALB/c mice (as complement source) and different dilutions of heat‐inactivated pooled sera from AIRmin, AIRmax or BALB/c mice immunized with PCV13 (six mice per group), were added to the wells in a final volume of 50 μL. Plates were incubated for 45 min at 37°C and 5% CO_2_ with constant shaking. Reactions were stopped by incubation on ice for 20 min. Samples were diluted 1:10 in OBB and 5 μL were plated on THY agar plates and incubated at 37°C for 15 h for CFU counting. As controls, bacteria were incubated in the presence of cells and complement source, without the addition of immune sera. Sera from AIRmin, AIRmax and BALB/c mice inoculated with saline were used as immunization controls. Phagocytosis was considered significant when a 50% reduction in the number of CFU was observed. Results were obtained with sera from two immunization experiments.

OPKA was also performed with purified bone marrow‐derived neutrophils from AIRmin, AIRmax and BALB/c mice (four per group). Bone marrow cells were extracted from mice femurs, in 3 mL of PBS with the aid of a syringe. Purification of neutrophils was performed by negative enrichment using the Neutrophil Isolation Kit (MACS–Miltenyi Biotec, USA), according to manufacturer instructions. After the protocol, neutrophils in all samples corresponded to at least 95% of the cells. OPKA was performed as described above.

### 2.8 Whole Blood Phagocytosis Assay

Neutrophils phagocytic capacity was evaluated as described [31]. Briefly, citrated peripheral blood from mice (five per group) was incubated with carboxymethylated beads (Kisker Biotech, Germany) coated with mice IgG (Sigma Aldrich, USA), calibrator (Alexa 405‐SE–Invitrogen, USA) and reporter (Oxyburst Green H2DCFDA‐SE ‐Invitrogen) fluorophores for 0‐, 15‐ and 60‐min. Cells were then stained with Live‐Dead (Fixable Far Red Dead Cell Stain Kit–Invitrogen), anti‐Ly6G‐APC‐Cy7 (BD Biosciences, clone 1 A8) and anti‐CD11b–PerCP Cy5.5 (BD Biosciences, clone M1/70). Erythrocytes were lysed with FACS lysing buffer (BD Biosciences). Samples were analyzed by flow cytometry in a FACS Canto II equipment (BD Biosciences) with 20,000 live neutrophils acquired. Phagocytosis was evaluated by the MFI of the Oxyburst Green H2DCFDA‐SE in live Ly6G^+^ CD11b^+^ AF405^+^ cells and the phagocytic index (PI) was calculated as follows: PI = (number of neutrophils with beads associated/number of total neutrophils) × OR, where OR (oxidative ratio) = Oxyburst MFI/AF405 MFI. Data were analyzed with FlowJo 10.1 software. Results were confirmed in three independent assays with blood from different mice (*n* = 4 per experiment).

### 2.9 Statistics

One‐way ANOVA with Tukey's post‐test was used for the analysis of anti‐PS antibodies, bacterial loads and neutrophil numbers. For comparisons of neutrophil phagocytic capacity, Two‐way ANOVA with Tukey's post‐test was applied. Survival was analyzed using the Log‐Rank survival curve with Mantel‐Cox for comparison between groups. In all cases, *p* ≤ 0.05 was considered significant. Graphs and statistical analyzes were performed with Prism GraphPad 9.0 (Dotmatics, USA).

## 3 Results

### 3.1 PCV13 Protects Airmin Mice Against Infection With ST1 but Not ST3 Pneumococci

**Figure 1:**
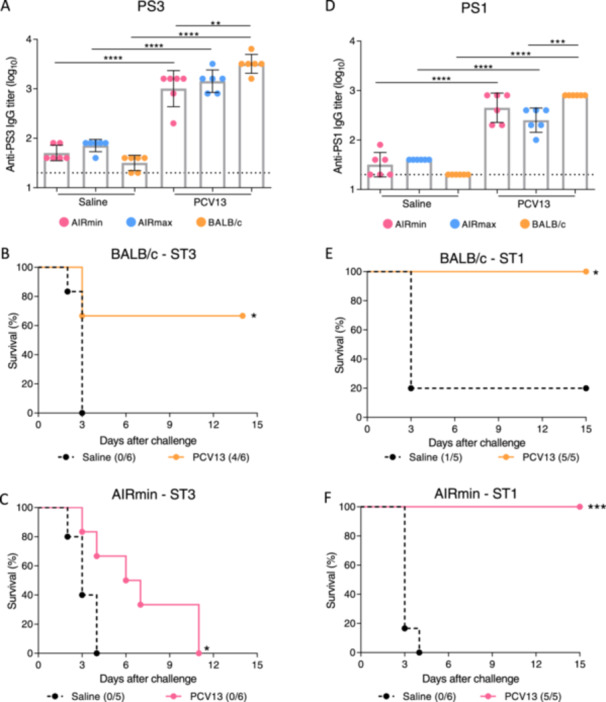
PCV13 antibody responses and protection against pneumococcal infection. Anti‐PS3 (A) and anti‐PS1 (D) IgG levels were evaluated by ELISA in sera obtained 14 days after the third immunization of AIRmin, AIRmax and BALB/c mice (six per group) with PCV13 or saline. Dots indicate individual data, and bars indicate the means for each group with standard deviations. Dashed line indicates the limit of detection. Results are representative of three independent experiments. Survival curves of immunized AIRmin and BALB/c mice (5–6 per group) after the challenges with ST3 (B and C) or ST1 (E and F) pneumococcal strains. Survival was analyzed for 15 days. **p < 0.01; ***p < 0.001; ****p < 0.0001, One‐way ANOVA with Tukey post‐test (A and D) and Log‐Rank survival curve with Mantel‐Cox test, B, C, E and F).

AIRmin, AIRmax and BALB/c mice were immunized subcutaneously with three doses of PCV13 or saline as a control. High levels of anti‐PS3 IgG (Figure 1A) were induced by immunization, in the three mice lines. BALB/c presented slightly higher levels of anti‐PS3 IgG, that were significant when compared to AIRmin mice. Such higher levels were consistently observed in different experiments, as can be observed in Supporting Information S1: Figure S1. Twenty‐1 days after the third immunization, mice were subjected to a lethal respiratory challenge with the 3JYP2670 strain, a ST3 pneumococcal strain. As expected, the ST3 strain was highly virulent in BALB/c and AIRmin mice, with all animals in the saline control groups dying by 4 days after infection (Figure 1B,C). Immunization with PCV13 conferred significant protection to BALB/c mice, with 66% of mice surviving the challenge (Figure 1B). For AIRmin mice, immunization with PCV13 increased the time to death, but 100% of the animals died within 11 days after infection (Figure 1C). As previously described by our group, AIRmax mice are highly resistant to pneumococcal infection [25] and even the control group survived the infection with the ST3 strain (Supporting Information S1: Figure S2). Since the increased ability of ST3 to evade vaccine‐induced immunity has been described [18, 19], we evaluated whether immunization with PCV13 could protect AIRmin mice against invasive disease caused by another serotype, using a ST1 strain (ATCC6301). High levels of anti‐PS1 IgG were observed in the sera of AIRmin, AIRmax and BALB/c mice immunized with PCV13, with BALB/c mice showing, once again, slightly higher antibody levels, mainly when compared to AIRmax mice (Figure 1D). AIRmin and BALB/c mice were then challenged with the ATCC6301 strain and protection was evaluated. Immunization with PCV13 conferred survival to 100% of both BALB/c (Figure 1E) and AIRmin mice (Figure 1F) against the challenge with the ST1 strain, demonstrating the high efficacy of this vaccine in this invasive model. Due to the previously observed resistance of AIRmax mice to pneumococcal infections [25] and Supporting Information S1: Figure S1, challenge of these mice with the ST1 pneumococcal strain was not performed. The resistance of AIRmax mice to pneumococcal infections shows that these mice are not adequate models to study the efficacy of pneumococcal vaccines, although some aspects of specific immune responses, such as the induction of anti‐PS antibodies can be observed. In summary, the high levels of anti‐PS1 and anti‐PS3 IgG induced by immunization of AIRmin mice with PCV13 were sufficient to confer survival of these mice against ST1 but not against ST3 pneumococci.

### 3.2 Colonization of the Respiratory Tract by ST3 Pneumococci is not Reduced in Airmin Mice Vaccinated With PCV13

**Figure 2:**
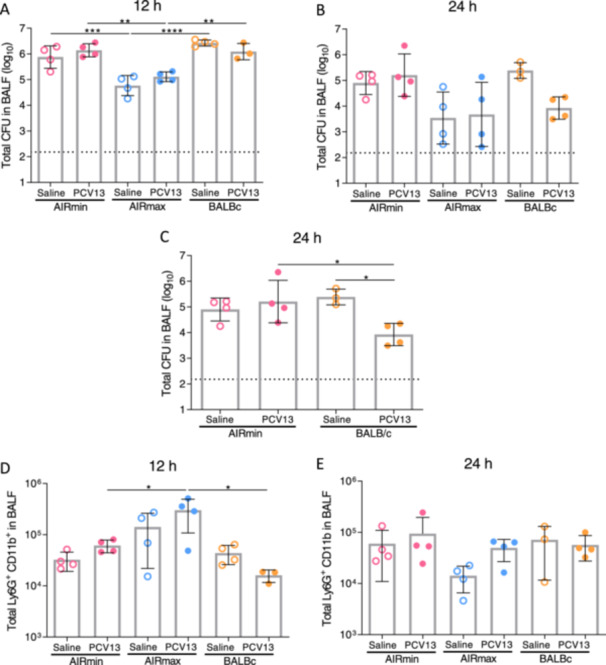
Bacterial colonization and neutrophil influx in the respiratory tract of mice immunized with PCV13. Mice (3–4 per group) received three doses of PCV13 or saline and were challenged 21 days after the last dose with the ST3 pneumococcal strain. BALFs were collected at 12 h (A and D) or 24 h (B, C and E) for the evaluation of bacterial colonization (A–C) or neutrophil influx (D and E). Neutrophil detection was performed by flow cytometry as the Ly6G^+^ CD11b^+^ population in 30,000 events counted. Data were analyzed with the FlowJo V10.1 software. Dots indicate individual data, and bars indicate the means for each group with standard deviations. Dashed lines indicate the limits of detection. *p < 0.05; **p < 0.01; ***p < 0.001; ****p < 0.0001, One‐way ANOVA with Tukey posttest.

We then compared the number of bacteria in the respiratory tract of mice vaccinated with PCV13 and challenged with ST3 pneumococci. Mice were inoculated intranasally with the 3JYP2670 strain 21 days after the third immunization, and BALFs were collected at 12 and 24 h post‐challenge. Both AIRmin and BALB/c mice displayed high numbers of bacteria in BALF collected at 12 h after the challenge, with no differences between control or vaccinated groups (Figure 2A). Conversely, lower numbers of bacteria were observed in AIRmax mice at 12 h postinfection, when compared to BALB/c and AIRmin mice in both vaccinated and control groups (Figure 2A). This result agrees with the natural resistance of AIRmax mice to pneumococcal invasive infection. At 24 h postchallenge, high numbers of bacteria are still observed in AIRmin mice, even in the group immunized with PCV13. A trend towards the reduction in the number of bacteria was observed in BALB/c mice vaccinated with PCV13, in comparison with the respective saline control group (Figure 2B). Once again, in both non‐vaccinated and PCV13‐vaccinated AIRmax mice, a lower number of bacteria was observed at 24 h after the challenge (Figure 2B). Since the effect of PCV13 vaccine in protection against pneumococcal infection cannot be evaluated in AIRmax mice, the same data were analyzed again, comparing only BALB/c and AIRmin mice (Figure 2C). We observed a significant reduction in pneumococcal numbers in BALFs collected from BALB/c vaccinated with PCV13, in comparison with the respective control as well as with AIRmin mice immunized with PCV13 (Figure 2C). These results suggest that the immune responses induced by PCV13 are not sufficient to promote bacterial clearance in the respiratory tract of AIRmin mice.

The number of neutrophils in the respiratory tract of mice, after pneumococcal challenge, were evaluated by flow cytometry (gating analysis presented in Supporting Information S1: Figure S3). BALB/c and AIRmin mice showed similar numbers of neutrophils at 12 h after the challenge, independent of vaccination with PCV13 (Figure 2D). At this time‐point, AIRmax mice displayed slightly higher numbers of neutrophils, with significant differences in the group vaccinated with PCV13, when compared to AIRmin or BALB/c, mice (Figure 2D). These results agree with the capacity of these mice to rapidly clear bacteria from the lungs. Variations in the numbers of neutrophils were observed at 24 h after the challenge, with no significant differences among the groups (Figure 2E).

### 3.3 Sera From Vaccinated Airmin Mice Have Reduced Capacity to Induce Pneumococcal Opsonophagocytosis In Vitro

PCV induce antibodies that opsonize pneumococcus, facilitating phagocytosis and clearance. We characterized and compared the anti‐PS3 antibodies from AIRmin, AIRmax and BALB/c mice immunized with PCV13. Anti‐PS3 IgG subclasses in sera were compared, but no significant differences were observed among the mice lines. PS3‐IgG1 was the isotype with highest levels, followed by IgG3 and IgG2a/2c and IgG2b in sera from the three mouse lines (Supporting Information S1: Figure S4).

**Figure 3:**
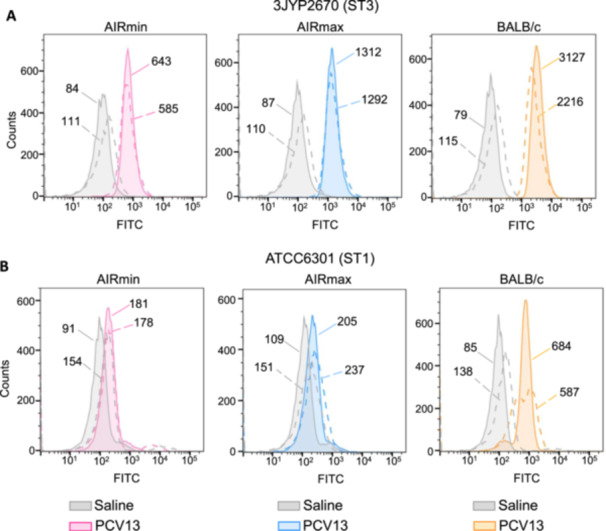
IgG binding to the surface of ST3 and ST1 pneumococcal strains. Pneumococcal strains ATCC6301 (ST1) or 3JYP2670 (ST3) were incubated with pooled sera from mice immunized with three doses of PCV13 or saline, followed by incubation with anti‐mouse IgG conjugated to FITC. Binding was detected by flow cytometry with 15,000 events recorded. Data were analyzed with the FlowJo V10.1 software and graphs were produced with data from two experiments using sera from independent immunization experiments. Solid lines represent one experiment and dashed lines represent the other. All data were produced using 1% sera except experiment 2 (dashed line) for ATCC6301 that used 5% sera. Numbers indicate the MFI for each curve.

The capacity of IgG to bind to pneumococcal surface in vitro was evaluated in flow cytometry assays, using FITC‐conjugated anti‐mouse IgG and ST1 and ST3 pneumococcal strains. The MFI of the curves were compared. Sera from all mice immunized with PCV13 exhibited IgG binding when compared with the respective saline controls (Figure 3). However, IgG in AIRmin sera displayed reduced capacity to bind to the ST3 surface, with MFIs of 643 and 585, using two different pooled sera, when compared to AIRmax sera (MFIs = 1312 and 1292) or BALB/c sera (MFI = 3127 and 2216) (Figure 3A). This reduced capacity was also observed for the ST1 strain, with MFIs of 181 and 178 produced by AIRmin sera, compared with MFIs of 205 and 237 produced by AIRmax sera and 684 and 587 produced by BALB/c sera (Figure 3B).

**Figure 4:**
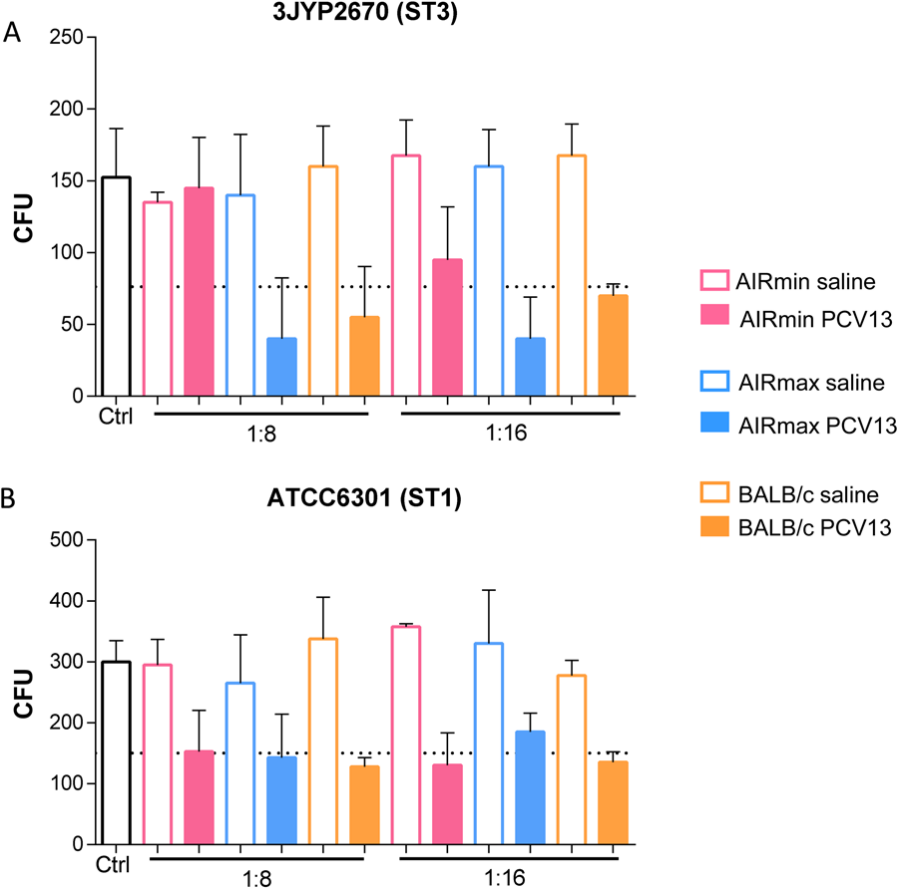
Induction of pneumococcal opsonophagocytosis by sera from mice immunized with PCV13. Sera from AIRmin, AIRmax and BALB/c mice (6 per pooled sera) immunized with three doses of PCV13 or saline were tested in opsonophagocytosis assays using J774 cells and ST3 (A) or ST1 (B) pneumococcal strains. Controls (Ctrl) contain all reaction components (cells, complement source and bacteria) except immune sera. Incubations were performed for 45 min and phagocytosis was considered significant when 50% reductions in CFU counting in relation to the control were observed (dashed line). Bars represent the mean with standard deviations. Graphs were composed with sera from two independent immunization experiments.

Sera were also compared for their ability to induce phagocytosis of pneumococci in vitro, by murine J774 macrophages (Figure 4). In the two dilutions tested (1:8 and 1:16), sera from AIRmin mice immunized with PCV13 were not able promote a reduction in the numbers of the ST3 pneumococcal strain, 3JYP2670, to at least 50% of the experimental control, which was the minimal limit considered for phagocytic activity (Figure 4A). On the other hand, both sera from AIRmax and BALB/c mice promoted opsonophagocytosis of the ST3 strain, in the two dilutions tested (Figure 4A). The lower opsonophagocytosis activity of the AIRmin sera was confirmed using another ST3 pneumococcal strain, the A66.1 strain (Supporting Information S1: Figure S5). Once again, in the two dilutions tested, sera from AIRmin mice showed low capacity to induce opsonophagocytosis of the ST3 pneumococcal strain, whereas positive results were observed for sera from AIRmax and BALB/c mice (Supporting Information S1: Figure S5). We then compared the capacity of the sera to induce phagocytosis of the ATCC6301 (ST1) strain. Sera from AIRmin mice immunized with PCV13 were able to promote phagocytosis of the ST1 pneumococcal strain, with comparable results with sera from AIRmax and BALB/c mice (Figure 4B). Together, these results indicate that the levels of anti‐PS antibodies induced in AIRmin mice can produce significant phagocytosis of ST1 but not ST3 pneumococci in vitro.

**Figure 5:**
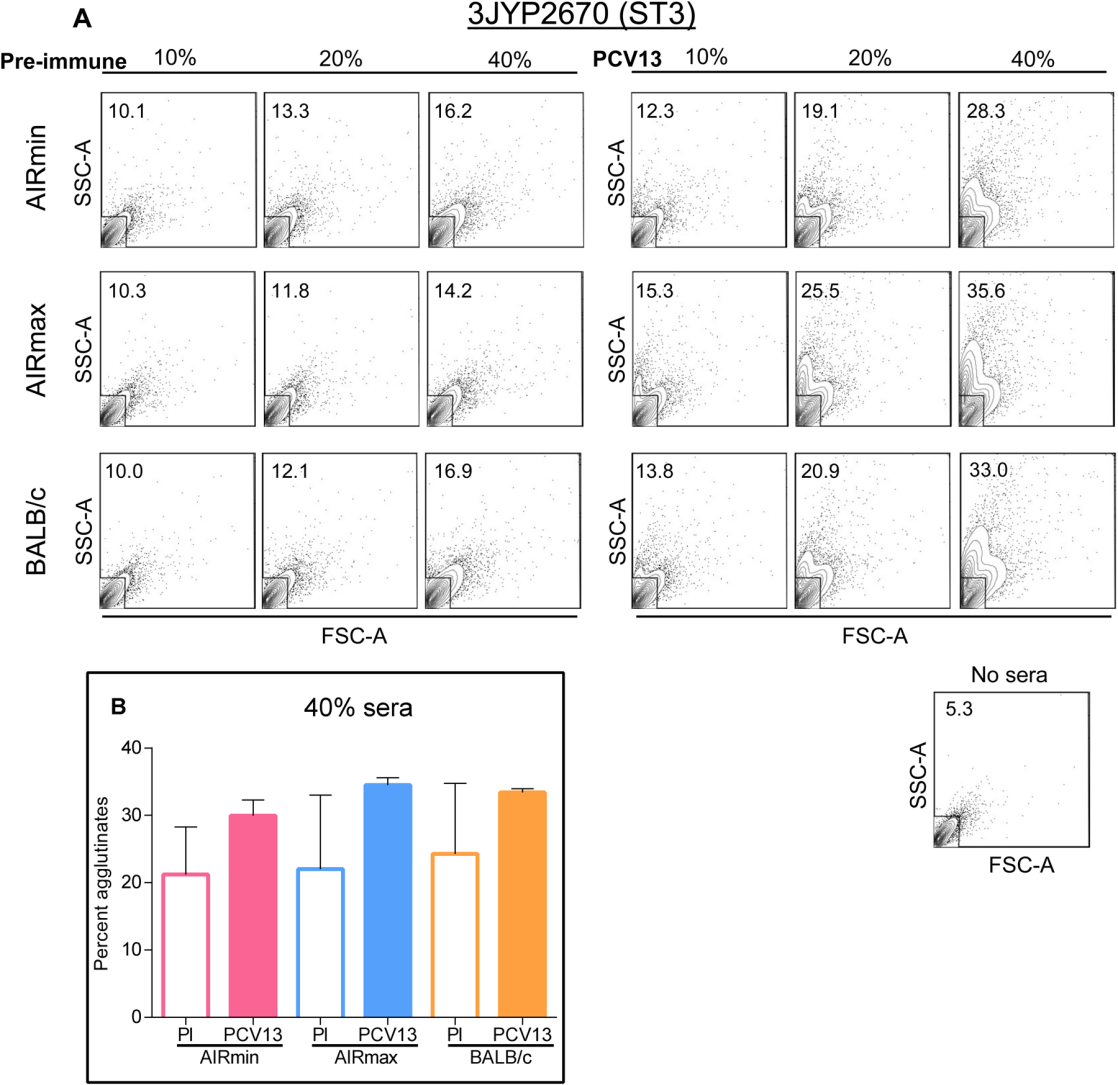
Agglutination of ST3 pneumococci by sera from mice (six per pooled sera) immunized with PCV13. The 3JYP2670 pneumococcal strain was incubated with different concentrations of pooled sera from AIRmin, AIRmax and BALB/c mice immunized with three doses of PCV13 or pre‐immune sera. Bacterial populations were selected based on forward scatter (FSC) and side scatter (SSC) parameters, with the acquisition of 200,000 events and data were analyzed with FlowJo V10.1 software. The numbers indicate the percentage of bacterial agglutinates. Results are representative of two assays with sera from independent immunization experiments (A). Sera from three independent pools (at a concentration of 40%) were compared for the ability to induce pneumococcal agglutination (B).

**Figure 6:**
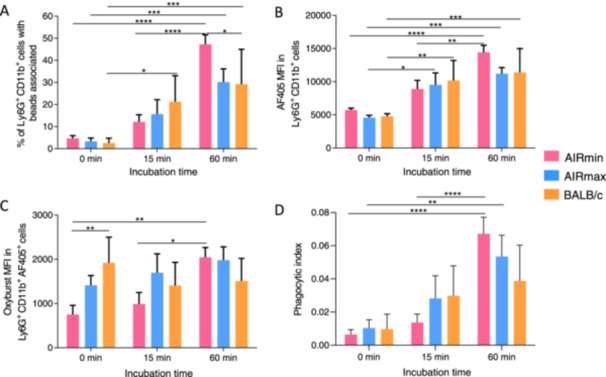
Phagocytic activity of circulating neutrophils from AIRmin, AIRmax and BALB/c mice. Peripheral blood from AIRmin, AIRmax and BALB/c mice (four per group) was incubated with beads adsorbed with the calibrator (AF405) and reporter (Oxyburst) fluorophores for 0, 15 or 60 min. Neutrophils were selected by flow cytometry as the Ly6G^+^ CD11b^+^ population. The percentage of neutrophils with beads associated were defined as the Ly6G^+^ CD11b^+^ AF405^+^ cells (A). The MFI for AF405 was also analyzed (B). Oxydative burst was inferred by the MFI of the Oxyburst Green H2DCFDA‐SE fluorophore measured in Ly6G^+^ CD11b^+^ AF405^+^ cells (C) and by the Phagocytic Index (PI) (D). Flow cytometry was performed with 20,000 live neutrophils acquired and data were analyzed using FlowJo V10.1 software. Bars indicate mean for each group (*n* = 4) with standard deviations. Results are representative of three independent experiments.

Agglutination of pneumococci is an effect that can be induced in vitro by anti‐PS antibodies and that was related to the capacity of the immune sera to promote bacterial clearance in vivo [32, 33]. The ability of sera from AIRmin, AIRmax and BALB/c mice immunized with PCV13 to agglutinate the ST3 pneumococcal strain 3JYP2670 was evaluated by flow cytometry, characterizing the percentage of agglutinates by size and granularity, in the presence of increasing concentrations of sera. Pre‐immune sera were used as controls. All anti‐PCV13 sera induced the agglutination of ST3 bacteria when compared to the respective pre‐immune controls and in a dose‐dependent manner. Sera from AIRmin mice immunized with PCV13 showed slightly lower agglutination capacity of the ST3 strain 3JYP2670, especially at the concentration of 40% of the anti‐PCV13 sera (Figure 5A). Quantification of the percentage of agglutinated pneumococci was performed using three independent pooled sera and the results showed a slightly lower activity of the sera from AIRmin mice immunized with PCV13 when compared to the sera of BALB/c mice, although the differences were not statistically significant (Figure 5B). This lower capacity was better observed when the sera were tested against the ST3 pneumococcal strain A66.1 (Supporting Information S1: Figure S6). In this case, the highest concentration of anti‐PCV13 AIRmin sera (40%) produced equivalent percentage of pneumococcal agglutinates as 20% of anti‐PCV13 sera from AIRmax or BALB/c mice (Supporting Information S1: Figure S6A). Once again, quantification of pneumococcal agglutinates using 40% pooled sera confirmed the lower activity of anti‐PV‐13 AIRmin sera (Figure 6B). We did not observe agglutination of the ST1 pneumococcal strain, ATCC6301, with any of the sera tested, suggesting that this in vitro assay may not be applied for all pneumococcal ST or strains.

In summary, although PCV13 induces the production of IgG against PS3 in AIRmin mice, functionality of the immune sera in in vitro assays was reduced. This agrees with the reduced protective capacity of PCV13 against ST3 infection in AIRmin mice.

### 3.4 Neutrophils From AIRmin Mice Display Full Phagocytic Functions

The phagocytic capacity of neutrophils from AIRmin, AIRmax and BALB/c mice was compared using a flow cytometry assay with a reporter bead to detect phagosomal oxidative capacity in neutrophils [31, 34] (gating strategy presented on Supporting Information S1: Figure S7). Labeled beads were incubated with whole blood from the three mice lines and their fluorescence in neutrophils (Ly6G^+^ CD11b^+^ cells) were compared at different time‐points. In this experiment, settings were adjusted to acquire 20,000 live neutrophils from each sample to eliminate possible differences in neutrophils counts. The percentage of neutrophils with beads associated (AF405^+^ neutrophils) increases during the incubation period for all samples. At 15 min incubation, a significant increase in the percentage of neutrophils with beads associated was observed in BALB/c samples, in relation to the respective basal level. At 60 min incubation, the percentages of neutrophils with beads associated increased significantly in all samples (Figure 6A). AIRmin samples showed the highest percentage of neutrophils with beads associated at the later time‐point. The MFI for the control fluorophore (AF405) in the neutrophil populations also increased during the incubation period. A significant increase was observed in BALB/c and AIRmax samples after 15 min and in all samples after 60 min (Figure 6B). The MFI of the reporter Oxyburst fluorophore, which refers to the oxidative activity of phagosomes was lower in the neutrophils of AIRmin mice when compared with AIRmax and BALB/c, right after the incubation (0 min), but it increased significantly after 60 min of incubation (Figure 6C). For AIRmax samples, an increase in relation to the basal level was observed at 15 min and 60 min, although with no statistical significance, due to the high intensity already observed at the basal level (Figure 6C). Oxyburst MFI in BALB/c neutrophils were already very high at the basal level and remained high throughout the experiment (Figure 6C). Calculation of the phagocytic index (PI) showed a progressive increase along the incubation period for neutrophils from the three mice lines, with no significant differences among them (Figure 6D). Interestingly, some differences were observed while acquiring 20,000 live neutrophils for the experiment. For all samples, as the incubation time increased, higher numbers of total cells had to be acquired to get 20,000 live neutrophils, indicating cell death. However, in samples from AIRmax mice, these numbers were reached with the acquisition of less total cells, indicating that these mice display high numbers of neutrophils in blood (Supporting Information S1: Figure S8A). Further analyzes confirmed higher percentages of live neutrophils in blood samples of AIRmax mice when compared to BALB/c and AIRmin mice, particularly at the basal level (time point = 0 min) (Supporting Information S1: Figure S8B). Direct immunophenotypical evaluation of Ly6G^+^ CD11b^+^ cells by flow cytometry (Supporting Information S1: Figure S9) also confirmed the higher numbers of neutrophils in peripheric blood from AIRmax mice, with statistical differences to AIRmin mice (Figure S10A). Moreover, AIRmax mice also showed increased levels of the neutrophil chemoattractant CXCL5 in blood when compared to AIRmin and BALB/c mice (Supporting Information S1: Figure S10B). No differences were observed in the numbers of neutrophils in bone‐marrow (Supporting Information S1: Figure S10C). The presence of high percentages of circulating neutrophils in the blood of AIRmax mice may contribute to the resistance to pneumococcal infections.

**Figure 7:**
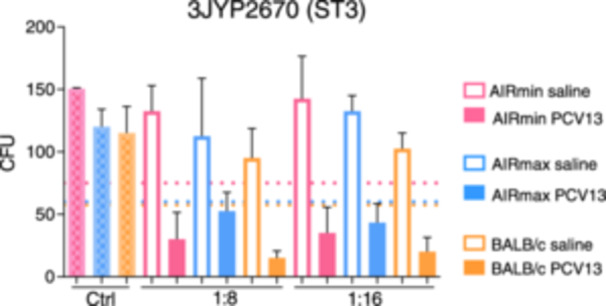
Opsonophagocytosis of ST3 pneumococci by neutrophils from AIRmin, AIRmax and BALB/c mice in the presence of PCV13‐immune sera. Bone‐marrow derived neutrophils from AIRmin, AIRmax and BALB/c mice (4 per group) were compared for their phagocytic capacity against ST3 pneumococci (3JYP2670 strain) in the presence of sera from mice immunized with three doses of PCV13 or saline. Controls (Ctrl) contain all reaction components (neutrophils from each mice line, complement source and bacteria) except immune sera. Incubations were performed for 45 min and phagocytosis was considered significant when 50% reductions in CFU counting in relation to the respective controls were observed (dashed lines) and plating was performed in duplicates. Bars represent the mean for each group (*n* = 4).

Finally, we compared the phagocytic capacity of bone‐marrow derived neutrophils from AIRmin, AIRmax and BALB/c mice, against the ST3 3JYP2670 pneumococcal strain, by OPKA assay. As controls, neutrophils from each mice line were incubated with bacteria and complement source in the absence immune sera. Therefore, three dashed lines representing the 50% reduction for each condition were produced. In addition, the corresponding saline or PCV13 sera from each mice line were used. The results showed that all neutrophils, including those derived from AIRmin mice, were highly effective in killing pneumococci, reducing the CFU counting below 50% in the presence of two different dilutions of the PCV13 immune sera (1:8 and 1:16) and at a cell: bacteria ratio of 400:1 (Figure 7). The high killing capacity of neutrophils observed in this assay, abrogated the inferiority of the AIRmin sera observed in the previous assays. These results thus confirm the capacity of neutrophils from AIRmin mice to kill pneumococci in vitro.

## 4 Discussion

PCV vaccines, which have been used in children globally since 2000, have greatly reduced the incidence of pneumococcal colonization and invasive disease caused by ST included in the formulations [18, 35]. The protective effects of PCV are also relevant in non‐vaccinated individuals, such as adults and the elderly, due to the reduction in circulation of vaccine‐type strains, resulting in herd‐immunity. Nevertheless, pneumococci remain as one of the most important pathogens causing respiratory infections and the leading cause of deaths associated with these infections [2]. The increase in the incidence of disease caused by ST that are not covered by the current PCV formulations contributes to this scenario [18, 19]. However, a special condition is observed for ST3 pneumococci, which is included in the PCV13. PCV13 indeed reduced the incidence of pneumococcal diseases caused by ST3 [35], but a relatively high occurrence of invasive diseases caused by this ST is still observed, suggesting a rate of escape from vaccine‐induced immunity [36, 37].

AIRmin and AIRmax mice, phenotypically selected for low and high acute inflammatory responses, respectively, have been used in studies of cancer [38], ulcerative colitis [39], arthritis [40] and envenomation by snake venoms [41]. Both mice lines were also previously compared in their susceptibility to infection with the intracellular bacteria *Lysteria monocytogenes* and *Salmonella thyphimurium*, with AIRmax mice being significantly more resistant than AIRmin mice [38, 42].

Inflammation is an essential host response for the elimination of bacteria during infection. In the case pneumococci, the model of lung infection in mice show that local inflammatory responses in the first hours are important for bacterial clearance. However, the thick PS capsule provides an advantage to ST3 pneumococci to evade host innate responses, resulting in longer permanency of the bacteria in the lungs. In this condition, the inflammatory stimulus may result in tissue damage with the spreading of bacteria to the bloodstream and the worsening of animal health conditions [43]. Using a protein‐based vaccine, we have shown that protection against ST3 infection in BALB/c mice correlated with a strong inflammatory response in the lungs at 12 h postinfection and a rapid decrease in inflammation after this period [26]. The influence of inflammation on the outcome of ST3 infection was also previously explored by us using AIRmin and AIRmax mice. Secretion of several inflammatory cytokines in the lungs in the first hours after infection was similar between the two mice lines, but AIRmin mice was not able to clear the bacteria, resulting in prolonged inflammation and increased susceptibility to ST3 infection [25]. In the line with these previous results, this study aimed to evaluate the protective potential of the PCV13 vaccine against ST3 infection in AIRmin mice as well as to characterize the immune response induced in this model.

PCV13 induced high levels of anti‐PS3 and anti‐PS1 IgG in both AIRmin and AIRmax mice, with no significant differences between them. These results show that the differential inflammatory status of these mouse lines did not result in differences in the magnitude of antibody responses to PS3 or PS1. However, the induction of anti‐PS3 and anti‐PS1 was consistently higher in BALB/c mice, in different experiments. Further, IgG in BALB/c sera had the highest capacity to bind to the surface of ST3 and ST1 pneumococci, whereas IgG from AIRmin mice showed the lowest binding capacity. These results correlate with the lack of protection against ST3 observed in AIRmin mice vaccinated with PCV13. On the other hand, despite the lower capacity of the IgG from AIRmin sera to bind to the surface of ST1 pneumococci, protection was observed. Thus, the relatively lower antibody response in AIRmin mice affected protection only against ST3 pneumococci. The ST3 capsule is attached to the pneumococcal surface through noncovalent interactions. Choi and collaborators have shown that ST3 pneumococcal strains release significantly higher concentrations of capsular PS (CPS) to the supernatant during in vitro growth than ST1, 4, 6B and 14. In addition, higher release of CPS3 was observed in the blood of mice infected with a ST3 strain through the intraperitoneal route [44] and sera from mice infected with ST3 contained free CPS3 that inhibited opsonophagocytic killing of pneumococci by an anti‐PS3 sera. Moreover, filtered supernatants of ST3 growth inhibited protection of anti‐PS3 antisera in passive immunization experiments in mice. The amount of ST3 culture supernatant required to significantly reduce protection in mice was 100x lower than the amount of ST4 culture supernatant to produce a similar effect against protection induced by an anti‐PS4 antisera [44]. The results described by Choi and collaborators provide a mechanism by which ST3 bacteria evade the antibody response, showing that the high concentrations of CPS3 released by ST3 pneumococci during infection compete for the anti‐PS3 antibodies induced by vaccines and support the hypothesis that protection against ST3 requires higher levels of antibodies.

Immunization of BALB/c mice with PCV13 led to a reduction in the bacterial numbers in the lungs 24 h after the challenge in comparison to nonvaccinated mice, showing the protective effect of the vaccine against the pulmonary infection in the murine model. However, immunization of AIRmin mice did not promote the same effect and bacterial numbers remained high at 24 h postchallenge. We evaluated the presence of anti‐PS3 IgG or IgA in BALF samples, at 12 h or 24 h after the challenge, but these antibodies were not detected, in the conditions tested.

The binding capacity of IgG from the sera of AIRmax mice vaccinated with PCV13 to the surface of ST3 and ST1 was intermediate between the binding capacity of sera from AIRmin and BALB/c mice. Since AIRmax mice are naturally resistant to pneumococcal infections, including ST3, it is impossible to conclude any role of the antibodies on protection of these mice. Bacterial numbers in the respiratory tract were lower than the observed for the other mice, regardless of vaccination. Thus, innate immune responses in AIRmax mice are efficient in promoting pneumococcal clearance, as previously described by our group [25].

In agreement with the lower protective capacity of PCV13 in AIRmin mice, sera from vaccinated AIRmin mice showed reduced capacity to induce opsophagocytosis of two ST3 pneumococcal strains (3JYP2670 and A66.1) when compared with sera from AIRmax and BALB/c mice. Conversely, sera from vaccinated AIRmin mice were as effective as AIRmax or BALB/c immune sera in inducing opsonophagocytosis of ST1 pneumococci, further corroborating the in vivo protection studies. In addition to the reduced opsonophagocytic activity, sera from AIRmin mice immunized with PCV13 displayed lower levels of IgG binding to pneumococcal surface and also showed a slightly reduced capacity to agglutinate ST3 pneumococci in vitro, which has been proposed as a mechanism that facilitate bacterial clearance [32, 33].

The profile of anti‐PS3 IgG subclasses in the sera of AIRmin, AIRmax or BALB/c mice vaccinated with PCV13 was similar. Therefore, evaluation of IgG subclasses in polyclonal sera did not provide any apparent relationship with the failure of PCV13 protection in AIRmin mice. Using a monoclonal antibody against PS3 (IE2), Doyle and collaborators described an effect in bacterial gene expression that resulted in decreased resistance of a ST3 to oxidative stress. The IE2 antibody did not induce opsonophagocytosis of ST3 pneumococci in vitro, but protected mice against nasopharyngeal colonization [45]. An additional study, using monoclonal antibodies against PS3, derived from B cells isolated from humans vaccinated with PCV13, also confirmed these properties [29]. These works provide insights for monoclonal therapies against pneumococcal infections and show the complexity of antibody effects against ST3.

The phenotypic selection of AIRmin and AIRmax mice was based on the protein concentrations and the numbers of cells in local infiltrates after subcutaneous inoculation of a neutral substrate (biogel beads) [24]. Further characterization of these mouse lines showed that AIRmax mice display a higher number of neutrophils in the blood when compared to AIRmin and BALB/c mice. Upon biogel stimulation, 85% of cells present in local exudates of AIRmax mice were neutrophils and these cells were more resistant to apoptosis [46]. Despite these quantitative differences, no differences in the expression of surface markers such as CD11b, CD62L or Gr1 and in PMA‐induced oxidative burst were observed, when the neutrophils of AIRmax mice were compared to AIRmin or BALB/c mice [46]. In a previous study we observed lower numbers of infiltrated neutrophils and lower concentrations of the CXCL5 chemokine in BALFs of AIRmin mice, at 12 h after pneumococcal infection, when compared to AIRmax mice [25]. In addition, AIRmin mice presented a higher percentage of infiltrated neutrophils in apoptosis or necrosis after pneumococcal infection, suggesting that a reduced neutrophil function could potentially account for the AIRmin increased susceptibility to infection [25]. Here we confirmed that AIRmax mice have higher numbers of circulating neutrophils and also observed higher concentrations of the neutrophil chemoattractant CXCL5 in the blood, which may contribute for its natural resistance to pneumococcal infection. We also compared the phagocytic capacity of neutrophils using a bead‐based assay and whole blood samples from AIRmin, AIRmax and BALB/c mice. No significant differences were observed in the oxidative capacity of the neutrophils among the three mouse lines, showing that the increased susceptibility of AIRmin mice to pneumococcal infection seems not to be due to an impairment in neutrophil function. This experiment also showed that AIRmax mice contained more live neutrophils in blood samples. Thus, the higher numbers of circulating neutrophils or local infiltrated neutrophils in AIRmax mice may contribute to pneumococcal clearance, resulting in natural resistance to infection. Finally, we compared the opsonophagocytic killing capacity of bone‐marrow derived neutrophils against ST3 pneumococci in vitro. In the conditions tested, all neutrophils, including those derived from AIRmin mice were able to kill the bacteria.

In summary, we have shown a difference of PCV13 efficacy against infection with ST3 and ST1 pneumococci in a mouse model selected for low acute inflammatory responses. The lack of protection against ST3 infection in AIRmin mice correlated with the induction of significantly lower levels of anti‐PS3 antibodies when compared to BALB/c mice and lower in vitro functionalities of the sera regarding IgG binding to bacterial surface and induction of opsonophagocytosis. Our results highlight the necessity of an optimized antibody response for protection against ST3 pneumococci. The AIRmin and AIRmax murine models also support the concept that both hosts and bacterial factors influence the outcome ST3 pneumococcal infection.

## Author Contributions

**Giuliana S. Oliveira:** data curation, formal analysis, investigation, methodology, writing–original draft, writing–review and editing. **Johanna Rivera:** data curation, methodology, writing–review and editing. **Tasson C. Rodrigues:** methodology, writing–review and editing. **Giovanna B. Carneiro:** methodology, writing–review and editing. **Orlando G. Ribeiro:** formal analysis, writing–review and editing. **Eliane N. Miyaji:** data curation, formal analysis, writing–review and editing. **Liise‐anne Pirofski:** conceptualization, formal analysis, funding acquisition, supervision, writing–original draft, writing–review and editing. **Maria Leonor S. Oliveira:** conceptualization, data curation, formal analysis, funding acquisition, investigation, project administration, supervision, writing–original draft, writing–review and editing.

## Conflicts of Interest

The authors declare no conflicts of interest.

## Supporting information

Supporting information.

## Data Availability

All data will be fully available upon request.
