# Supplementary material for: Serotype 3 Streptococcus pneumoniae Escapes the Immune Responses Induced by PCV13 in Mice With High Susceptibility to Infection
Source: Immun Inflamm Dis. 2024 Dec 6;12(12):e70062. doi: 10.1002/iid3.70062 (PMC11621863; doi:10.1002/iid3.70062)
Supplement: Supplementary file 1 — Supporting information. [file IID3-12-e70062-s001.docx]

**Supplemental material**

ELISA for detection of anti-PS3 IgG subtypes

ELISA was performed as described in Materials and Methods, with minor differences. After incubation with mice immune sera, goat anti -IgG1 -IgG2a, IgG2c, IgG2b or IgG3 (Southern Biotech, USA) were added to the wells followed by incubation with HRP conjugated anti-goat IgG. Reactions were developed as described in Materials and Methods.

ELISA for detection of CXCL5

CXCL5 detection was performed in sera from non-immunized mice using the DuoSet®ELISA kit (R&D systems, USA), according to the manufacturer's instructions. Absorbances were read in an EZ microplate reader 400 (Biochrom, USA) at 450 nm, discounting the absorbances at 595 nm. The results were expressed in concentrations based on a CXCL5 standard curve.

Bone marrow and blood neutrophils counting

Cell counting was performed in bone marrow and blood from mice. Citrated blood (100 µL) was diluted in 120 µL of PBS. Bone marrow was extracted from femurs, in 3 ml of PBS with the aid of a syringe. Cells (1x10^6^) were suspended in PBS containing 1:100 dilutions of the anti-Ly6G - APC-Cy7 (BD Biosciences, clone 1A8) and anti-CD11b – PerCP Cy5.5 (BD Biosciences, clone M1/70) antibodies. Samples were incubated for 30 min, at 4 ºC in the dark and fixed with 100 µL Cytofix (BD Biosciences). The acquisition of 50,000 events was carried out on a FACS Canto II equipment (BD Biosciences) and results were analyzed using the FlowJo V10.1 software.

**Figure S1.** Anti-PS3 IgG induced by immunization of mice with PCV13. IgG levels were evaluated by ELISA in sera obtained 14 days after the third immunization of AIRmin, AIRmax and BALB/c mice with PCV13. Dots indicate individual data, and lines indicate the means for each group. Dashed line indicates the limit of detection. Each dot color represents an individual immunization experiment. *P<0.05; ***P<0.001, One-way ANOVA with Tukey post-test.

**Figure S2.** Survival of AIRmax mice against pneumococcal infection. Mice were immunized with three doses of PCV13 or saline. Invasive challenge was performed with the ST3 pneumococcal strain and survival was monitored for 15 days. Log-Rank survival curves were produced using the Prism GraphPad.

A B C


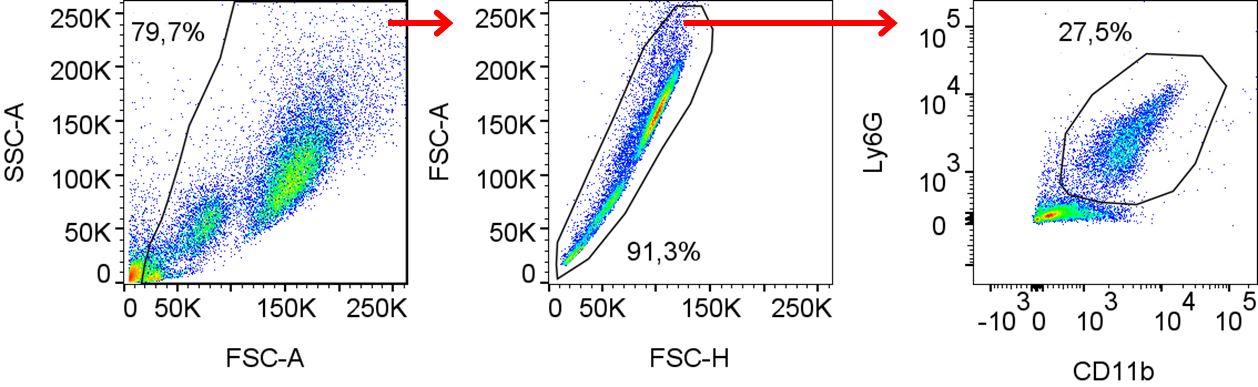


**Figure S3.** Analysis of neutrophils in BALF samples. Infiltrated neutrophils were assessed in BALF from AIRmin, AIRmax and BALB/c mice immunized with PCV13 at 12 and 24 h after the challenge with ST3 pneumococcal strain (3JYP2670). Cell debris were excluded by size and granularity using the side scatter (SSC-A) and forward scatter (FSC-A) parameters (A), followed by the elimination of cell aggregates B). Neutrophil populations were defined as the Ly6G^+^ CD11b^+^ cells (C). Plots from a representative sample of AIRmin mice are shown. Data were analyzed using the FlowJo V10.1 software.

**Figure S4.** Anti-PS3 IgG subclasses induced by vaccination of mice with PCV13. The titers of anti-PS3 IgG isotypes were evaluated by ELISA in sera obtained 14 days after the third immunization of AIRmin, AIRmax and BALB/c mice with PCV13. Bars indicate the means for each group (n=6) with standard deviations. Dashed line indicates the limit of detection. Results are representative of two independent experiments.

**Figure S5.** Induction of pneumococcal opsonophagocytosis by anti-PCV13 sera. Sera from AIRmin, AIRmax and BALB/c mice immunized with three doses of PCV13 or saline were tested in opsonophagocytosis assay using J774 cells and the ST3 A66.1 pneumococcal strain. Control (Ctrl) contains all reaction components (cells, complement source and bacteria) except immune sera. Incubations were performed for 45 min, with 1:16 and 1:40 dilutions of immune sera, and phagocytosis was considered significant when 50% reductions in CFU counting in relation to the control were observed (dashed line). Bars represent the mean of duplicates with standard deviations. Graphs were composed with sera from two independent immunization experiments.

**A**


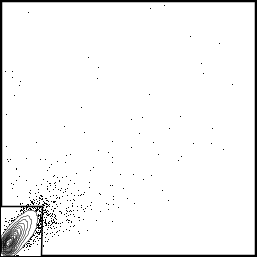


5.6

No sera

SSC-A

FSC-A


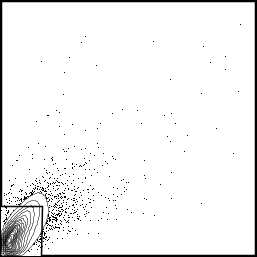


11.6


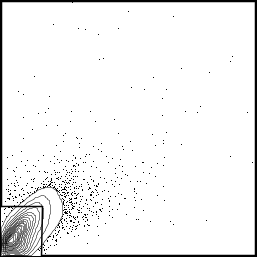


16.5


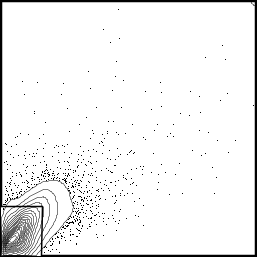


20.9


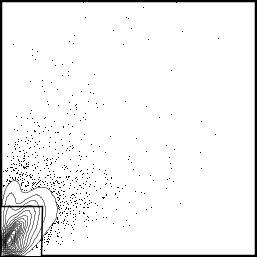


16.6


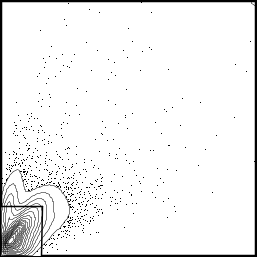


20.6


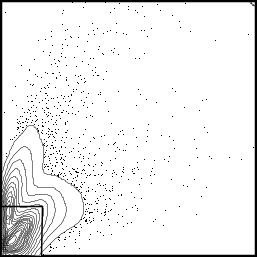


34.5


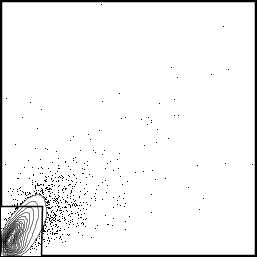


10.7


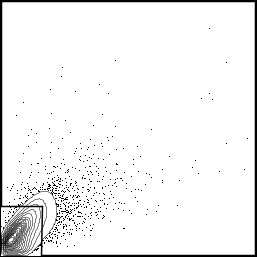


14.1


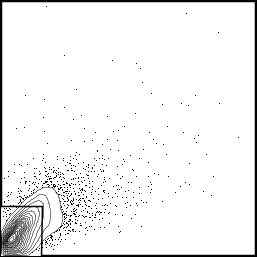


16.6


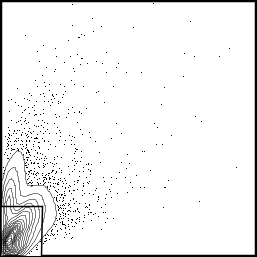


21.5


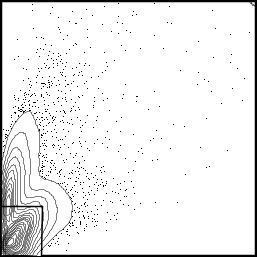


35.4


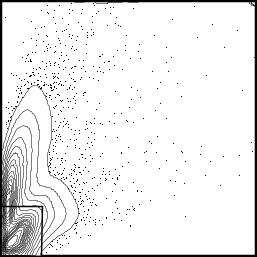


47.5


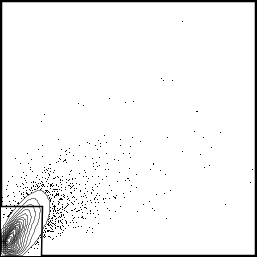


12.4


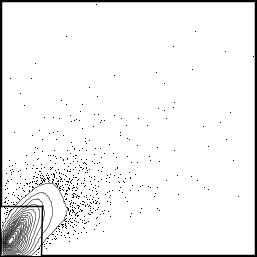


17.8


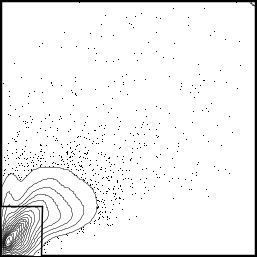


29.0


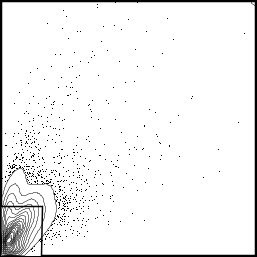


19.1


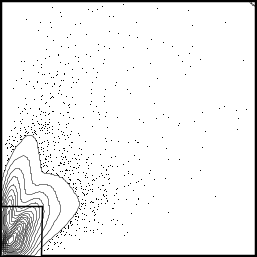


30.3


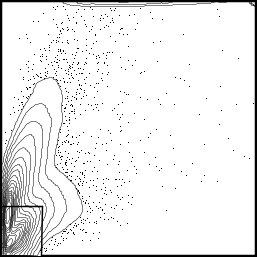


51.8

AIRmin

**PCV13**

**Pre-immune**

AIRmax

BALB/c

SSC-A

A66.1 (ST3)

10%

20%

40%

FSC-A

10%

20%

40%

SSC-A

SSC-A

FSC-A

**Figure S6.** Agglutination of ST3 pneumococci by sera from mice immunized with PCV13. The A66.1 pneumococcal strain was incubated with different dilutions of pooled sera from AIRmin, AIRmax and BALB/c mice immunized with three doses of PCV13 or pre-immune sera. Bacterial populations were selected based on forward scatter (FSC) and side scatter (SSC) with the acquisition of 200,000 events and data were analyzed with FlowJo V10.1 software. The numbers indicate the percentage of bacterial agglutinates. Results are representative of two assays with sera from independent experiments (A). Sera from three independent pools (at a concentration of 40%) were compared for the ability to induce pneumococcal agglutination (B).


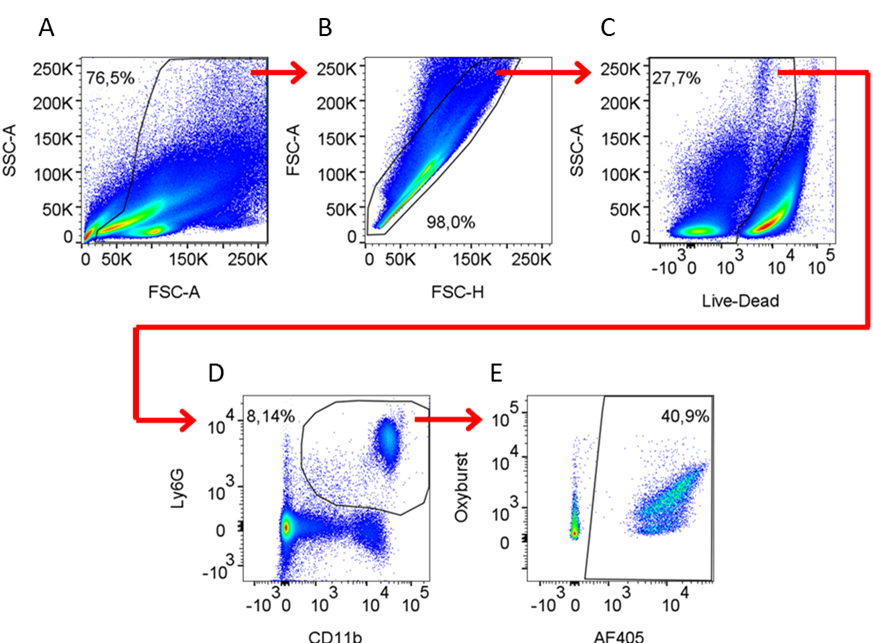


**Figure S7.** Strategy for the analysis of phagocytic activity in peripheral blood- neutrophils. Peripheral blood from AIRmin, AIRmax and BALB/c mice was incubated with beads adsorbed with calibrator (AF405) and reporter (Oxyburst^TM^) fluorophores for 0, 15 or 60 min. Cell debris were excluded by size and granularity using the side scatter (SSC-A) and forward scatter (FSC-A) parameters (A), followed by the elimination of cell aggregates (B). Selection of live cells was performed using the live/dead stain (C) and neutrophils were defined as the Ly6G^+^ CD11b^+^ cells (D). Neutrophils with beads associated were defined as the Ly6G^+^ CD11b^+^ AF405^+^ (E). The mean fluorescence intensities of the AF405 and Oxyburst^TM^ curves were analyzed in this population, normalized for 20,000 live neutrophils. Plots from a representative sample of AIRmin mice are shown.

 A


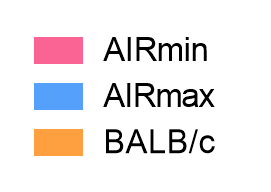

B

**Figure S8.** Evaluation of neutrophil population in peripheral blood from AIRmin, AIRmax and BALB/c mice, during the phagocytosis assay. Peripheral blood from AIRmin, AIRmax and BALB/c mice was incubated with beads adsorbed with calibrator (AF405) and reporter (Oxyburst^TM^) fluorophores for 0, 15 or 60 min. Live neutrophils were selected by flow cytometry as the Ly6G^+^ CD11b^+^ population after selection with live/dead markers. Numbers of total cells acquisition to get 20,000 live neutrophils were compared (A) as well as the percentage of live neutrophils in total cells (B). Flow cytometry was performed with 20,000 live neutrophils (Ly6G^+^ CD11b^+^ acquired) and data were analyzed using FlowJo V10.1 software. Bars indicate mean for each group (n=4) with standard deviations. *P< 0.05 and ***P< 0.001, Two-way ANOVA with Tukey’s post-test. Results are representative of three independent experiments.

A B C


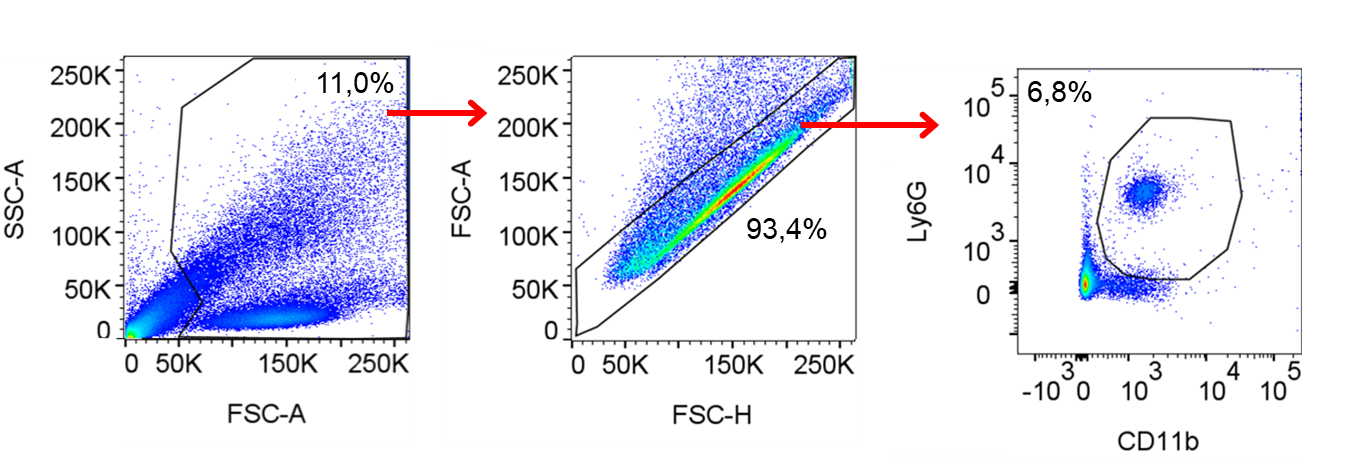


Blood

D E F


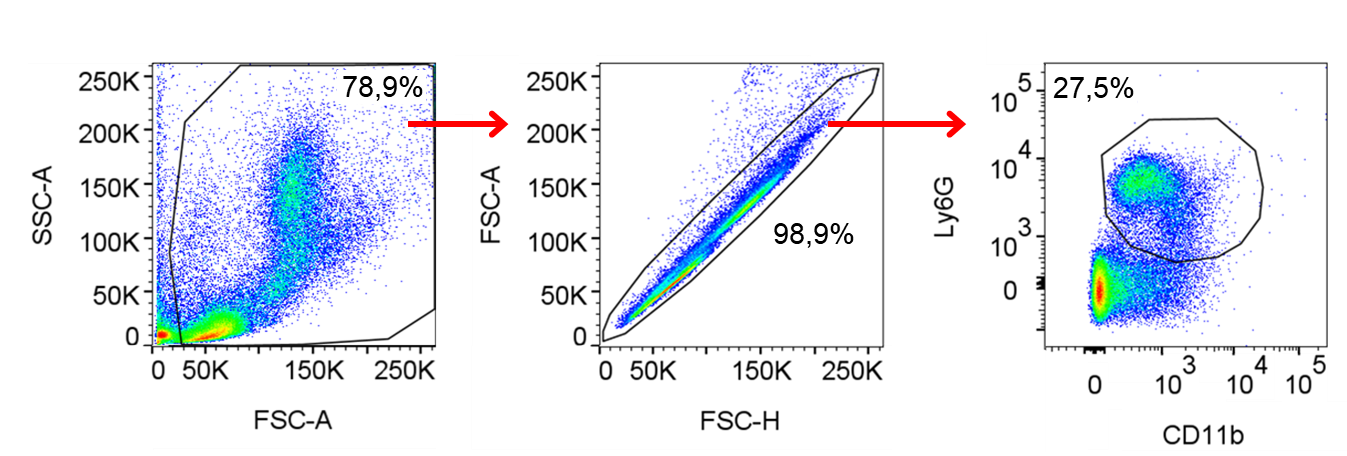


Bone marrow

**Figure S9.** Analysis of neutrophils in bone marrow and blood. Neutrophils were assessed in blood and bone marrow samples from naïve AIRmin, AIRmax and BALB/c mice. Cell debris were excluded by size and granularity using the side scatter (SSC-A) and forward scatter (FSC-A) parameters (A and D), followed by the elimination of cell aggregates (B and E). Neutrophil populations were defined as the Ly6G^+^ CD11b^+^ cells (C and F). Data were analyzed using the FlowJo V10.1 software. Plots from a representative sample of AIRmin mice are shown.

**
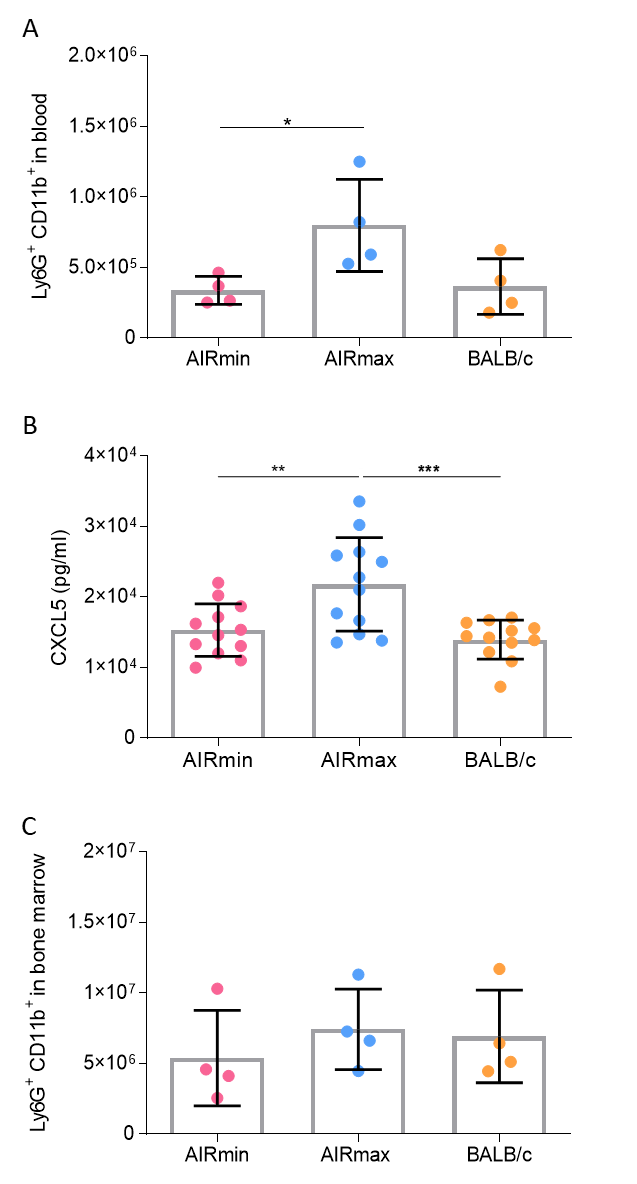
**

**Figure S10.** Basal numbers of neutrophils in blood and bone marrow and CXCL5 in sera of AIRmin, AIRmax and BALB/c mice. Blood (A and B) and bone marrow (C) were collected as described in materials and methods. (A and C) Neutrophil detection was performed by flow cytometry as the Ly6G^+^ CD11b^+^ population in 30,000 events acquired. Data were analyzed with the FlowJo V10.1 software. (B) CXCL5 chemokine was detected by ELISA in sera from non-immunized mice (1:1000 dilution). Dots indicate individual data, and bars indicate the means for each group with standard deviations. *P<0.05, **P< 0.01; ***P< 0,001, One-way ANOVA with Tukey post-test.
